# Association between exposure to ambient air pollution and renal function in Korean adults

**DOI:** 10.1186/s40557-018-0226-z

**Published:** 2018-02-28

**Authors:** Hyun-Jin Kim, Jin-young Min, Yong-Seok Seo, Kyoung-bok Min

**Affiliations:** 1 Institute of Health and Environment, Seoul National University, Seoul, 08826 Republic of Korea; 2 Institute of Health and Environment, Seoul National University, Seoul, 08826 Republic of Korea; 3 Institute of Environmental Research, Kangwon National University, Chuncheon, 24341 Gangwon-do Republic of Korea; 4 Department of Preventive Medicine, College of Medicine, Seoul National University, 103 Daehak-ro, Jongno-gu, Seoul, 03080 Republic of Korea

**Keywords:** Ambient air pollution, Association, Renal function, Korean adults

## Abstract

**Background:**

Ambient air pollution has a negative effect on many diseases, such as cardiovascular and respiratory diseases. Recent studies have reported a relationship between air pollution and renal function, but the results were limited to exposure to particulate matter (PM). This study was to identify associations between various air pollutants and renal function among Korean adults.

**Methods:**

Nationwide survey data for a total of 24,407 adults were analyzed. We calculated the estimated glomerular filtration rate (eGFR) for each individual to assess their renal function and used this to categorize those with chronic kidney disease (CKD). To evaluate exposure to ambient air pollution, we used the annual mean concentrations of four ambient air pollutants: PM with an aerodynamic diameter ≤ 10 μm (PM_10_), nitrogen dioxide (NO_2_), sulfur dioxide (SO_2_), and carbon monoxide (CO).

**Results:**

We identified significant inverse relationships between the air pollutants PM_10_ and NO_2_ and eGFR in all statistical adjustment models (all *p* < 0.05). In the full covariate model, interquartile range increases in the annual mean concentrations of PM_10_ and NO_2_ were associated with decreases in eGFR levels of 0.46 (95% CI = − 0.87, − 0.04) and 0.85 (95% CI = − 1.40, − 0.30), respectively. Three of the ambient air pollutants were significantly related to an increased risk of CKD in the unadjusted model (*p* < 0.0001), but all significant associations disappeared after adjusting for covariates (all *p* > 0.05).

**Conclusions:**

Exposures to PM_10_ and NO_2_ were significantly associated with decreases in eGFR levels, but not CKD, in Korean adults.

## Background

Ambient air pollution has recently been recognized as one of the most serious issues worldwide. According to World Health Organization (WHO) data, more than 90% of the world’s population lives in places that do not meet the WHO standards for air quality. The number of deaths due to outdoor air pollution is estimated to be about 3 million a year. Exposure to air pollution increases the risk of developing various diseases, including cardiovascular disease (CVD), chronic obstructive pulmonary disease, type 2 diabetes mellitus, and autoimmune rheumatic diseases [1–4]. The adverse effects of air pollution on cardiovascular health, in particular, are well established [1, 3, 5].

Recent studies have focused on understanding the relationship between renal function and air pollution [6, 7] because a rapid decline in renal function or chronic kidney disease (CKD) is closely linked to cardiovascular events and the cardiovascular mortality [8, 9]. Several researchers have hypothesized that exposure to air pollution in the form of particulate matter (PM) influences renal function via mechanisms similar to those proposed for CVD, such as inflammation or oxidative stress, given that renal dysfunction is a crucial risk factor for CVD [6, 7, 10]. Indeed, few studies have demonstrated the negative effects of air pollution on renal function indicators such as estimated glomerular filtration rate (eGFR) and CKD [6, 7].

Results from studies on the relationship between air pollutant exposure and renal function have been inconsistent. Previous reports have focused primarily on exposure to PM air pollution, represented by PM with an aerodynamic diameter of ≤ 10 μm (PM_10_) or ≤ 2.5 μm (PM_2.5_); no evidence has been reported regarding the association of renal function with other pollutants. Considering that exposure to other pollutants, such as nitrogen dioxide (NO_2_), sulfur dioxide (SO_2_), carbon monoxide (CO), and black carbons significantly associated with various diseases including CVD [3, 5, 11, 12], an integrated analysis of air pollutants for renal function is important.

The aim of this study, therefore, is to investigate whether ambient air pollution from PM_10_, NO_2_, SO_2_, and CO is related to renal function indicators in a nationwide sample of Korean adults. We evaluated eGFR and CKD status of each individual and analyzed the associations between these renal function indicators and exposure to the four air pollutants.

## Methods

### Study population

The data were obtained from the Korean National Health and Nutrition Examination Survey (KNHANES) conducted by the Korean Centers for Disease Control and Prevention. The KNHANES is a nationwide cross-sectional epidemiologic survey with a probability-cluster, multistage, and stratified sampling design. A total of 41,347 individuals participated in the 2007–2011 KNHANES. Of these, individuals who met the following criteria were excluded: (1) those under 30 years of age, (2) those lacking information about the administrative divisions needed to identify their air pollution exposure, (3) those without the phenotypic information needed for the eGFR calculation, such as age, sex, and serum creatinine level, and (4) those who reside in Jeju Island, which is an island region that is environmentally different from land area. Finally, a total of 24,407 adults were included in the statistical analysis. Written consent was obtained from all individuals before participating in the survey and their data were anonymized. This study was approved by the institutional review board of the Seoul National University Hospital Biomedical Research Institute.

### Renal function measurement

Blood samples were drawn from survey participants after fasting for at least 8 h. The serum creatinine level of each sample was measured by a professional blood testing agency. The individual’s eGFR, a representative value indicating renal function, was calculated using the Modification of Diet in Renal Disease (MDRD)-4 equation: GFR (mL/min per 1.73 m^2^) = 175 x SCr^-1.154^ x age^-0.203^ × 1.212 (if the individual was black) × 0.742 (if female), where SCr is the serum creatinine level. The CKD was defined as eGFR < 60 mL/min/1.73 m^2^, which represents a reduction in renal function of half or more of the normal level [13].

### Air pollution exposure

To estimate each individual’s exposure to ambient air pollution, we used monitoring data for 24-h concentrations of ambient air pollution collected from January 1, 2007, to December 31, 2011, by the Ministry of the Environment of Korea (https://www.airkorea.or.kr). These data were obtained from about 300 atmospheric monitoring sites nationwide in South Korea. The ambient air pollutants analyzed in the present study were PM_10_, NO_2_, SO_2_, and CO. The KNHANES survey data do not provide the actual home addresses of the survey participants, required to estimate the concentrations of air pollution, to which they were exposed. We therefore calculated the annual average concentrations of air pollutants for the 16 administrative divisions of South Korea (7 metropolitan cities and 9 provinces). Of these, one province (Jeju Island), which differs from the other administrative divisions environmentally and culturally, was excluded from this study. The individuals’ residential division codes were then used to link them to the annual average pollutant levels in 15 administrative divisions.

### Potential covariates

We investigated potential covariates for the associations between ambient air pollution and renal function from the KNHANES survey. Demographic data, including age, sex, household income, education level, smoking status, and alcohol consumption, were obtained via a questionnaire. Smoking status was coded as three categories: never-smoker, former-smoker, and current-smoker. Alcohol consumption was assessed according to the frequency of drinking alcohol per month over the previous year and was classified into four categories: never, less than once a month, twice or three times a month, and more than 4 times a month. We divided the daily protein intake (g) into four levels using quartiles, and the fourth quartile group (protein intake > 81.7 g) was defined as high protein intake group. Residential regions were classified into two categories: urban and rural. Anthropometric data such as height and weight were also obtained, and body mass index (BMI) was calculated as the weight in kilograms divided by the square of the height in meters (kg/m^2^). Clinical data for this study, including total cholesterol and fasting glucose, were obtained from blood. Diabetes was defined as fasting glucose ≥126 mg/dL or taking diabetes treatment medication or taking insulin by injection, or physician diagnose. Systolic blood pressure (SBP) and diastolic blood pressure (DBP) were measured three times, and the mean values of the second and third measurements were used in the analysis. Hypertension was defined as SBP ≥ 140 or DBP ≥ 90 mmHg or an individual taking hypertensive drugs for more than 20 days a month.

### Statistical analysis

We used Pearson’s correlation analysis to assess the correlations between the ambient air pollutants. Independent sample t-tests were conducted to evaluate the difference in annual mean air pollution levels between the two types of residential areas (urban and rural), as well as to compare the annual mean air pollution levels between CKD and normal groups. Multiple linear regression analysis was performed to identify associations between ambient air pollution and eGFR level, with the results indicated as beta coefficients and 95% confidence intervals (CIs) for renal function. The associations between ambient air pollution and CKD were determined by multiple logistic regression analyses, estimating the odds ratios (ORs) and 95% CIs of each air pollutant for CKD. These statistical estimates were scaled to the interquartile range (IQR) for each pollutant (10 μg/m^3^ for PM_10_, 12 ppb for NO_2_, 1 ppb for SO_2_, and 0.1 ppm for CO). We estimated three statistical models using gradually adjusted methods: unadjusted model (without covariates); Model 1, adjusted for age, sex, household income quartile, education level, smoking, alcohol consumption, high protein intake, survey year, and residential region; and Model 2, adjusted for Model 1 plus BMI, total cholesterol, fasting glucose, diabetes, systolic blood pressure, and hypertension. All analyses were performed with SAS 9.3 (SAS Institute, Cary, NC, USA).

## Results

The detailed characteristics of the study population are shown in Table 1. A total of 24,407 subjects were included in the association analyses. The mean age was 52.8 years, with a higher proportion of female (57.1%) than male (42.9%). The educational level of the subjects was relatively evenly distributed, and about 25.6% graduated from college or graduate school. The percentages of former and current smokers were 20.8% (*n* = 5023) and 21.2% (*n* = 5122), respectively. Approximately 70% of the subjects drank more than once a month and 8.1% drank more than four times a month. A much higher proportion of subjects lived in urban regions (65.1%) than in rural regions (34.9%). The mean waist circumference and BMI were 82.1 cm and 23.8 kg/m^2^, respectively. The mean SBP and DBP were 120.5 mmHg and 77.1 mmHg, respectively, with 34.2% of the subjects classified as hypertension (*n* = 8299). The mean eGFR calculated by MDRD-4 equation was 84.4 mL/min/1.73 m^2^, and 5.6% of the subjects were classified as having CKD (*n* = 1361).

**Table 1:** Characteristics of the study subjects (*n* = 24,407)

Characteristics	Mean ± SD or N (%)	Missing
Age (years)	52.8 ± 14.3	0
Female gender	13,933 (57.1)	0
Education level		299
≤ Elementary school	7425 (30.8)	
Middle school	3020 (12.5)	
High school	7484 (31.0)	
≥ College or graduate school	6179 (25.6)	
Smoking		250
Never	14,012 (58.0)	
Former-smokers	5023 (20.8)	
Current-smokers	5122 (21.2)	
Alcohol Consumption (per month)		313
Never	7382 (30.6)	
≤ 1	6659 (27.6)	
2–3	8108 (33.7)	
≥ 4	1945 (8.1)	
Daily protein intake (g)	66.0 (36.3)	2725
Residential region		0
Urban	15,881 (65.1)	
Rural	8526 (34.9)	
WC (cm)	82.1 ± 9.6	132
BMI (kg/m^2^)	23.8 ± 3.2	128
SBP (mm Hg)	120.5 ± 17.8	19
DBP (mm Hg)	77.1 ± 10.7	19
Hypertension^a^	8299 (34.0)	
Fasting Glucose (mg/dL)	99.1 ± 24.3	43
Diabetes^b^	2635 (10.8)	
Total cholesterol (mg/dL)	190.8 ± 36.4	50
Creatinine (mg/dL)	0.9 ± 0.2	0
eGFR mL/min/1.73 m^2c^	84.4 ± 17.1	
CKD^c^	1361 (5.6)	

*BMI* body mass index, *WC* waist circumference, *SBP* systolic blood pressure, *DBP* diastolic blood pressure, *eGFR* estimated glomerular filtration rate, *CKD* chronic kidney disease

^a^Hypertension was defined as a systolic blood pressure ≥ 140 mmHg or a diastolic blood pressure ≥ 90 mmHg or taking hypertension treatment medication more than 20 days a month

^b^Diabetes was defined as fasting glucose ≥126 mg/dL or taking diabetes treatment medication or taking insulin by injection or physician diagnose

^c^eGFR was calculated by MDRD-4 equation (GFR in mL/min per 1.73 m^2^ = 175 x SerumCr-1.154 x age-0.203 × 1.212 (if patient is black) × 0.742 (if female))

^c^CKD was defined as eGFR < 60 mL/min/1.73 m^2^

The median concentrations of PM_10_, NO_2_, SO_2_, and CO were 53 μg/m^3^, 24 ppb, 5 ppb, and 0.6 ppm, respectively, and their IQRs were 10 μg/m^3^, 12 ppb, 1 ppb, and 0.1 ppm, respectively (Table 2). All air pollutants were significantly inter-correlated (all *p* < 0.001), and the correlation between PM_10_ and CO was the strongest of these correlations (*r* = 0.63).

**Table 2:** Air pollutants (annual average concentrations) and their distributions

				Percentile					Pearson’s correlation coefficients			
Air pollutants	Mean	SD	IQR	10th	25th	50th	75th	90th	PM_10_	NO_2_	SO_2_	CO
PM_10_ (μg/m^3^)	52.5	5.8	10	46	47	53	57	60	1	0.52***	0.40***	0.63***
NO_2_ (ppb)	25.2	6.9	12	16	19	24	31	34	–	1	0.16***	0.34***
SO_2_ (ppb)	5.5	1.1	1	4	5	5	6	7	–	–	1	0.28***
CO (ppm)	0.6	0.1	0.1	0.4	0.5	0.6	0.6	0.7	–	–	–	1

*SD* standard deviation, *IQR* interquartile range, *PM*_*10*_ particulate matter < 10 μm in diameter, *NO*_*2*_ nitrogen dioxide, *SO*_*2*_ sulfur dioxide, *CO* carbon monoxide

^*^p < 0.05, ^**^p < 0.01, ^***^p < 0.001

Table 3 presents the results of the analysis of the associations between ambient air pollution and eGFR according to the adjustment models. In the results of the unadjusted model, all four air pollutants were significantly associated with decreased eGFR (all *p* < 0.001). However, after the adjustment for covariates (Model 1 and Model 2), SO_2_ and CO were no longer significantly associated with decreased eGFR (both *p* > 0.05). In the full covariate model (Model 2), the PM_10_ concentration was significantly associated with eGFR (*p* = 0.0314); with an IQR (10 μg/m^3^) increase in PM_10_ concentration, there was a 0.46 decrease in eGFR (95% CI = − 0.87, − 0.04). The eGFR was also significantly associated with the NO_2_ concentration (*p* = 0.0026); with an IQR (12 ppb) increase in NO_2_ concentration, there was a 0.85 decrease in eGFR (95% CI = − 1.40, − 0.30).

**Table 3:** Estimated associations of IQR increases in annual average air pollution and estimated glomerular filtration rate (eGFR)

	Unadjusted Model		Model 1^a^		Model 2^b^	
Air pollutants	β (95% CI)	*p*-value	β (95% CI)	*p*-value	β (95% CI)	*p*-value
PM_10_ (μg/m^3^)	−3.10 (− 3.47, − 2.73)	< 0.0001	− 0.58 (− 1.00, − 0.17)	0.0054	− 0.46 (− 0.87, − 0.04)	0.0314
NO_2_ (ppb)	− 0.78 (− 1.16, − 0.41)	0.0010	− 0.93 (− 1.47, − 0.39)	0.0008	−0.85 (− 1.40, − 0.30)	0.0026
SO_2_ (ppb)	−1.58 (− 1.78, − 1.38)	< 0.0001	0.14 (− 0.07, 0.34)	0.1903	0.17 (− 0.03, 0.38)	0.1015
CO (ppm)	−2.25 (− 2.48, − 2.01)	< 0.0001	−0.02 (− 0.27, 0.22)	0.8568	0.03 (− 0.21, 0.28)	0.7878

*CI*, confidence interval, *PM*_*10*_ particulate matter < 10 μm in diameter, *NO*_*2*_ nitrogen dioxide, *SO*_*2*_ sulfur dioxide, *CO* carbon monoxide

The beta coefficient and 95% confidence interval in each air pollutant was scaled to the interquartile range for each pollutant, respectively (10 μg/m^3^ for PM_10_, 12 ppb for NO_2_, 1 ppb for SO_2_, and 0.1 ppm for CO)

^a^Model 1 was adjusted for demographic variables including age, sex, household income quartile, education level, smoking, alcohol consumption, high protein intake, survey year, and residential region

^b^Model 2 was adjusted for demographic variables plus clinical variables including body mass index, total cholesterol, fasting glucose, diabetes, systolic blood pressure, and hypertension

The results of the assessment of the effects of ambient air pollution on CKD according to the three adjustment models are shown in Table 4. In the unadjusted model, the air pollutant concentrations except for NO_2_ were significantly associated with CKD (all *p* < 0.0001). In Model 1, the PM_10_ concentration was significantly associated with increased risk of CKD (OR = 1.14; 95% CI = 1.00, 1.29), but its statistical significance disappeared in the full adjusted model (Model 2) (*p* = 0.1665). In addition, no significant association between other air pollutants and CKD was observed in the adjusted model (Model 2 and Model 3).

**Table 4:** Estimated associations of IQR increases in annual average air pollution and chronic kidney disease (CKD)

	Unadjusted Model		Model 1^a^		Model 2^b^	
Air pollutants	OR (95% CI)	*p*-value	OR (95% CI)	*p*-value	OR (95% CI)	*p*-value
PM_10_ (μg/m^3^)	1.40 (1.27, 1.53)	< 0.0001	1.14 (1.00, 1.29)	0.0436	1.10 (0.96, 1.25)	0.1665
NO_2_ (ppb)	0.98 (0.89, 1.08)	0.6759	1.09 (0.93, 1.29)	0.2909	1.06 (0.89, 1.26)	0.5257
SO_2_ (ppb)	1.18 (1.13, 1.24)	< 0.0001	0.96 (0.91, 1.03)	0.2343	0.97 (0.91,1.04)	0.3535
CO (ppm)	1.26 (1.19, 1.34)	< 0.0001	1.02 (0.95, 1.09)	0.6420	0.99 (0.92,1.06)	0.7628

*OR* odds ratio, *CI* confidence interval, *PM*_*10*_ particulate matter < 10 μm in diameter, *NO*_*2*_ nitrogen dioxide, *SO*_*2*_ sulfur dioxide, *CO* carbon monoxide

The odds ratio and 95% confidence interval in each air pollutant was scaled to the interquartile range for each pollutant, respectively (10 μg/m^3^ for PM_10_, 12 ppb for NO_2_, 1 ppb for SO_2_, and 0.1 ppm for CO)

^a^Model 1 was adjusted for demographic variables including age, sex, household income quartile, education level, smoking, alcohol consumption, high protein intake, survey year, and residential region

^b^Model 2 was adjusted for demographic variables plus clinical variables including body mass index, total cholesterol, fasting glucose, diabetes, systolic blood pressure, and hypertension

## Discussion

This study investigated the relationship between ambient air pollution and renal function in Korean adults. We observed a significant inverse association of eGFR level with exposure to ambient air pollution, such as PM_10_ and NO_2_, showing 0.46 (95% CI = − 0.87, − 0.04) and 0.85 (95% CI = − 1.40, − 0.30) decrease in eGFR per each IQR increase in PM_10_ (10 μg/m^3^) and NO_2_ (12 ppb) concentration (Model 2). Ambient air pollution was also significantly associated with an increased risk of CKD in the unadjusted model (all *p* < 0.0001), but no significant association was observed after adjustment for all potential covariates (Model 2) (all *p* > 0.05). These results suggest that exposure to air pollution is significantly associated with decreased eGFR levels in Korean adults, but is less likely to lead to renal diseases such as CKD.

Previous epidemiological studies have identified significant associations between exposure to air pollutants and renal function [6, 7, 14]. In 2013, Lue et al. investigated the association of eGFR level with traffic-related air pollution exposure using residential proximity to major roadways in 1103 patients hospitalized for acute ischemic stroke. This showed significantly lower eGFR levels in patients living within 50 m of the nearest major roadway than in those living at least 1000 m away from the nearest major roadway [14]. More recent studies reported a relationship between ambient PM exposure and renal function. A longitudinal study in 669 older American men found that chronic exposure to ambient PM_2.5_ negatively influenced their renal function and was also associated with an increase in reduction in age-related eGFR level [6]. In addition, Yang et al. in 2016 evaluated the association between renal function and PM including PM10, coarse particles (PM_Coarse_), PM_2.5_ and PM_2.5Absorbance_ among Taiwanese adults, reporting significant associations of PM10 and PM_Coarse_ with renal function indicators including eGFR and CKD [7]. These previous findings regarding PM exposure and eGFR were in line with our results, although we did not evaluate PM_2.5_ due to absence of data. On the contrary, urinary albumin excretion, one of the indicators of renal function, showed no significant association with long-term exposure to PM_2.5_ or PM_10_ in the longitudinal analysis of the Multi-Ethnic Study of Atherosclerosis [10]. No significant association between PM_10_ concentration and CKD was observed in our study. The discrepancy in these results may be explained by differences in ethnicity, the indicators of renal function, the prevalence of CKD, the study region, the exposure assessment method, and covariates.

The present study demonstrated, for the first time, that exposure to NO_2_ was associated with decreased eGFR in Korean adults. This association was still significant after additional adjustment for PM_10_ concentration in the full covariates model (β = − 0.62; 95% CI = − 2.61, − 0.06) (Data not shown). As mentioned earlier, Lue et al.’s study identified the significant link between eGFR level and the distance to the nearest major roadway, an important source of NO_2_ emission [14]. A recent cross-sectional study also investigated the association between environmental exposure and uremic pruritus (UP), also known as chronic renal disease-related pruritus, among 866 patients undergoing hemodialysis. The researchers found that outdoor air pollution, including that from NO_2_ and CO, was closely related to UP [12]. In addition, short- and long-term exposure to NO_2_ has been shown to be significantly associated with respiratory and cardiovascular mortality [3, 5]. Although indirect evidence for the association of renal function with NO_2_ has been reported, there has been little direct evidence regarding the impact of NO_2_ exposure on renal indicators such as eGFR and CKD.

To date, the mechanisms underlying the link between ambient air pollution and renal function have not yet fully elucidated, but they may share the biological pathway proposed for the association between air pollution and CVD, given that reduced eGFR is a major risk factor for the development of CVD [9]. One potential physiological mechanism is the inflammatory response pathway. Exposure to air pollution has been shown to be closely related to increased levels of inflammatory biomarkers such as C-reactive protein, IL-6, IL-8, TNF-α, and fibrinogen [15–18]. The release of such pro-inflammatory cytokines contributes to the progressive decline in eGFR or the development of CKD and end-stage kidney disease [19, 20]. Alternatively, the relationship between ambient air pollution and renal dysfunction can be explained by oxidative stress pathway. Exposure to PM induces systemic oxidative stress via the increased production of reactive oxygen species in macrophages and endothelial cells or via air pollution-induced inflammation [21, 22]. Excessive production of reactive oxygen species is a major cause of endothelial cell injury or damage, and endothelial dysfunction is associated with renal failure [23]. Oxidative stress is also associated with a decrease in eGFR levels, as well as with the increased progression of CKD [22]. However, further research is needed to establish the mechanisms involved in air pollution and renal function.

Most previous studies of air pollution and renal function considered only exposure to ambient PM. The current study, based on large-scale national data for Korean adults, showed a negative effect of NO_2_ as well as PM_10_ on renal function. However, this study had some limitations that need to be considered. First, our study was conducted in cross-sectional study design; therefore, it is difficult to infer causality between exposure to ambient air pollution and renal function. The evidence for association in this design also may be weaker than that of cohort studies or real-time exposure studies. In addition, in such a study design, it is difficult to rule out the possibility that chronic kidney disease may be caused by other factors during long-term exposure. Second, we estimated the level of individuals’ exposure to ambient air pollution using only their residential region. This approach may not take into account the variability of individuals within the same administrative district (eg, job location, occupational exposure, daily activity area, indoor exposure time, residence period in the area, or distance to the main road at home), especially the metropolitan cities. However, in our study, a large sample size may be able to minimize these biases. Lastly, it is difficult to understand the precise duration of the previous exposure for each individual from the time of the survey, since the annual average concentrations of the year corresponding to the subject’s survey year due to were used in this study.

## Conclusions

Our study investigated the relationship between the air pollutants PM_10_, NO_2_, SO_2_, and CO and the kidney function indicators eGFR and CKD in Korean adults. This demonstrated that exposure to PM_10_ and NO_2_ were significantly associated with decreased eGFR levels, but not with CKD. Our findings provide evidence that exposure to the air pollutants PM_10_ and NO_2_ is closely associated with decreased eGFR level before the onset of renal disease.

## Abbreviations

PM_10_: PM with an aerodynamic diameter of ≤ 10 μm
PM_2.5_: PM with an aerodynamic diameter of ≤ 2.5 μm
BMI: Body Mass Index
CIs: Confidence Intervals
CKD: Chronic Kidney Disease
CO: Carbon Monoxide
CVD: Cardiovascular Disease
DBP: Diastolic Blood Pressure
eGFR: Estimated Glomerular Filtration Rate
IQR: Interquartile Range
KNHANES: Korean National Health and Nutrition Examination Survey
MDRD: Modification of Diet in Renal Disease
NO_2_: Nitrogen Dioxide
ORs: Odds Ratios
PM: Particulate Matter
SBP: Systolic Blood Pressure
SO_2_: Sulfur Dioxide
UP: Uremic Pruritus
WHO: World Health Organization
